# A Targeted Management of the Nutrient Solution in a Soilless Tomato Crop According to Plant Needs

**DOI:** 10.3389/fpls.2016.00391

**Published:** 2016-03-30

**Authors:** Angelo Signore, Francesco Serio, Pietro Santamaria

**Affiliations:** ^1^Department of Agricultural and Environmental Science, University of Bari Aldo MoroBari, Italy; ^2^Institute of Sciences of Food Production, National Research Council of ItalyBari, Italy

**Keywords:** water use efficiency, nutrient use efficiency, soilless, closed system, uptake concentration, environmental sustainability

## Abstract

The adoption of closed soilless systems is useful in minimizing the environmental impact of the greenhouse crops. Instead, a significant problem in closed soilless systems is represented by the accumulation of ions in the recycled nutrient solution (NS), in particular the unabsorbed or poorly absorbed ones. To overcome such problem, we: (1) studied the effect of several values of the electrical conductivity (EC) of NS in a NFT (Nutrient Film Technique) system on a cherry type tomato crop, and (2) define a NS (called recovery solution), based on the concept of “uptake concentration” and transpiration–biomass ratio, that fits the real needs of the plant with respect to water and nutrients. Three levels of EC set point (SP), above which the NS was completely replaced (SP5, SP7.5, and SP10 for the EC limit of 5, 7.5, and 10 dS m^-1^, respectively), were established. The SP10 treatment yield was not different from other treatments, and it allowed a better quality of the berries (for dry matter and total soluble solids) and higher environmental sustainability due to a lower discharge of total nutrients into the environment (37 and 59% with respect to SP7.5 and SP5, respectively). The recovery solution used in the second trial allowed a more punctual NS management, by adapting to the real needs of the crop. Moreover, it allowed a lesser amount of water and nutrients to be discharged into the environment and a better use of brackish water, due to a more accurate management of the EC of the NS. The targeted management, based on transpiration–biomass ratio, indicates that, in some stages of the plant cycle, the NS used can be diluted, in order to save water and nutrients. With such management a closed cycle can be realized without affecting the yield, but improving the quality of the tomato berries.

## Introduction

Tomato is the most important vegetable crop in the world (FAO, 2014)^1^. Its cultivation is widespread in the Mediterranean basin, in particular in the coastal zone. Unfortunately, the water used for crops irrigation in these zones is often scarce and of poor quality, because of the infiltration of brackish water into underground aquifers.

The soilless systems allow the use of brackish water without problems for the growing media and, furthermore, can help to decrease the consumption of water in greenhouses, because of more accurate management of fertigation (Bradley and Marulanda, 2000; Montesano et al., 2015). In tomato crops, the use of brackish water can even result in an improvement in the qualitative profile of the fruits (Adams and Ho, 1989; Adams, 1991; Petersen et al., 1998; Serio et al., 2004; Sato et al., 2006). Nevertheless, the environmental sustainability of open soilless systems has been questioned, because a more or less consistent fraction of nutrient solution (NS), is discharged into the environment (Sonneveld, 2002). This fraction varies largely in function of several parameters, but under normal growing conditions it ranges between 20 and 50%, although in some cases (e.g., at the beginning of the crop cycle or with low temperatures) this value can increase up to 80% (Grewal et al., 2011).

The adoption of closed soilless systems is useful in minimizing the environmental impact of the production process of greenhouse crops (Savvas, 2002). In such systems, the drainage percentage is not restricted by environmental concerns and hence the irrigation frequency may be considerably higher than that resulting in leaching fractions recommended for open cultivation systems. In closed systems the quantity of water provided to the crop, with respect to that theoretically required, is always higher resulting in several advantages (Silber et al., 2003).

A problem that should be taken into consideration in closed soilless systems is represented by the accumulation of ions, in particular the unabsorbed or poorly absorbed ones, a phenomenon which originates from higher ion to water inlet ratios (i.e., concentrations in the irrigation water) than the corresponding ion to water uptake ratios (Savvas et al., 2007a), resulting in an unbalanced ratio between nutrients and a higher EC in the NS.

To overcome such problems both automated sensing (Hak-Jin et al., 2013) and mathematical models have been developed (Silberbush et al., 2005; Savvas et al., 2007a; Massa et al., 2008). Although several of them are already available, their use is complicated by the fact that they need a lot of variables in order to work. Moreover, only a few models are suitable for greenhouse environments (Bacci et al., 2012), so they are often not suitable for a commercial application in greenhouses and, in some cases, their implementation is laborious because need to be adapted to the current situation of a crop (Gallardo et al., 2009; Bacci et al., 2012).

Starting from the above premises, the present study was designed to (i) verify the concentration Na^+^ level (and the related EC) in the NS without causing a decrease in yield or quality of a cherry type tomato crop grown with a closed soilless system and (ii) develop a NS (called recovery solution), based on the concept of “uptake concentration” (Sonneveld, 2002) and transpiration–biomass ratio, that fits the real needs of the plant with respect to water and nutrients.

## Materials and Methods

Two trials were carried out at the ‘La Noria’ Farm of the Institute of Sciences of Food Production of the National Research Council, Mola di Bari (41°03′N, 17°4′E – Apulia, Italy), in a polymethacrylate greenhouse with a maximum height of 4.5 m.

### Common Traits to Both Experiments

#### Crop System

In both trials, the plants were transplanted at the fourth true leaf stage, on 23 January for the first and 3 February for the second trial, using a hybrid cultivar of cherry tomato (*Solanum lycopersicum* L., cv. Naomi). Plants were arranged on eleven aluminum benches (length 6 m, width 0.26 m, 1% sloped); two external rows and two extra plants at the beginning and end of every row served as guards. All benches were covered with plastic sheeting, black colored on the underside and white on the upperside.

Plants were placed with a density of 3.3 plants⋅m^-2^, deriving from a distance between benches of 1.2 m, and a distance of plants into the bench equal to 0.25 m. The plants were trained vertically and topped at the 10th cluster, and periodic operations such as binding, lateral stem, and basal leaf pruning were carried out. Minimum temperatures inside the greenhouse were set to ≥15/13°C (day/night), whereas above 20°C the greenhouse temperature was controlled by natural ventilation through the automatic ridge openings. Pollination was guaranteed by the introduction into the greenhouse of bumblebees (*Bombus terrestris*).

#### Nutrient Solution Management and Water Consumption

Plants were grown with the Nutrient Film Technique (NFT, a soilless re-circulated closed system), and the NS was supplied over the whole benches using pumps (one for each bench). Every bench was served by a tank containing 100 L of NS. The fertigation was supplied discontinuously (50 min every hour) with a flux of 3–4 L⋅min^-1^. Every 2 days fresh NS was added to the tanks, up to the initial volume of 100 L, in order to replenish NS consumed. After the replenishment, the EC and pH of the NS were measured and the latter, where appropriate, corrected with H_2_SO_4_ or NaOH, in order to maintain the pH in the 5.5–6.5 interval.

The water consumption was calculated on alternate days by measuring the volume of NS added to each tank by means of a volume meter. The volume of NS discharged during the cycle or at the end of it was measured in the same way as the NS added. Fortnightly samples of NS were withdrawn from tanks, after replenishment with the fresh NS, in order to verify the level of the inorganic ions. A sample of NS was also withdrawn at the end of the crop cycle in order to verify the concentration of the residual nutrients not absorbed by the crop.

During the crop cycle, to avoid the excessive raising of the greenhouse temperature, the greenhouse was protected with a 50% shadow cloth from 116 days after transplant (DAT).

#### Yield and Quality

Harvest started 121 DAT and 115 DAT, and finished, together with crop cycle, 169 and 167 DAT, for the first and second trial, respectively. Fruits were harvested when 80% of the berries on the truss were completely red. The fruits were divided into marketable and discarded classes (i.e., those showing blossom-end rot or radial cracks on the epicarp). The fruits in the discarded class were counted, weighed and discarded. The fruits in the marketable class were numbered, separated into two size classes (diameter 25–35 mm and >35 mm) and weighed for the fresh mass.

In the first trial total soluble solids (TSS) and dry matter (DM) of the harvested fruits were determined for every truss. In the second trial DM was determined for every truss, while the TSS was only determined in one harvest.

#### Physical and Chemical Analysis

Inorganic ions (both from NS and plant material) were determined by ion chromatography (Dionex model DX500; Dionex Corporation, Sunnyvale, CA, USA) with a conductivity detector, using the pre-column IonPack AG14 and the column of separation IonPack AS14 for the anions, and the pre-column IonPack CG12A and the column of separation IonPack CS12A for the cations (Di Gioia et al., 2013). Ultrapure water at 18 MΩ/cm (Milli-Q Academic Millipore) was used in all the analysis.

Total Kjeldahl nitrogen in the plant was determined from 0.1 g of dried and ground leaf tissue by the Kjeldahl method (Kjeltec 2300 Auto Analyser; Foss-Tecator, Hillerød, Denmark) adding salicylic acid to recover the NO_3_-N (15 mL 0.18 M salicylic acid in 96% H_2_SO_4_, selenium compounds and Zn as catalysts). Plant phosphorus content was determined by spectrophotometry.

The pH and EC of NS were measured using the portable pH-meter HI 9025 and the conductivity-meter HI-9033 (Hanna Instruments, Padova, Italy). The pH was not subjected to analysis because its correction was done only to maintain its value in the 5.5–6.5 range, in the same manner for all the treatments.

Total soluble solids were measured using a portable reflectometer (Brixstix BX 100 H; Techniquip Corporation, Livermore, CA, USA); the DM was determined after drying until constant weight in a forced-draft oven at 65°C.

### Experimental Treatments

#### First Trial

**FIGURE 1:**
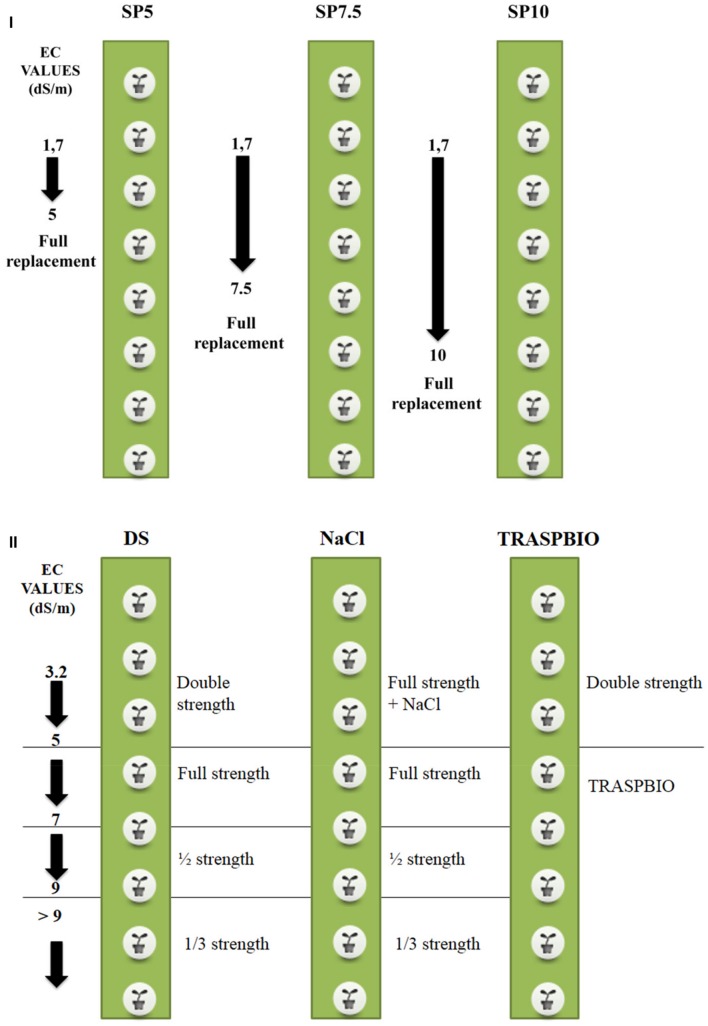
**Scheme of the experimental treatments and management of nutrient solution (NS) for the first (I) and second trial (II).** The arrows indicate the maximum value of EC reached for replacement of the NS.

The treatments were arranged in a completely randomized design with three replications, and they differed for the EC set point (SP) above which the NS inside the tanks was completely replaced, were the following (**Figure 1I**): (1) SP5: full replacement of the NS when the EC in the tanks exceeded 5 dS m^-1^; (2) SP7.5: full replacement of the NS when the EC in the tanks exceeded 7.5 dS m^-1^ and (3) SP10: full replacement of the NS when the EC in the tanks exceeded 10 dS m^-1^.

The starting value of the EC of the NS was 1.7 dS m^-1^ in all the treatments (**Figure 1I**).

The reaching of the EC value was verified after the addition of NS to the single tanks to the volume of 100 L.

The concentration of NS during the crop cycle is reported in **Table 1**.

**Table 1:** Concentration of the macro nutrients used in the nutrient solution (NS).

Phenological phases	N_NO_3_	N_N H_4_	K^+^	P	Mg^2+^	Ca^2+^	SO_4_^2-^-S
	(mM)						
Start of cycle - Fourth inflorescence	9.4	0.6	6.1	1.6	1.6	3.2	2.5
Fourth inflorescence - Beginning of harvest	10.1	0.6	7.7	1.6	1.9	3.2	0.7
Beginning of harvest – Third truss	8.1	0.5	7.7	1.6	1.9	2.5	0.4
Third truss – End of the cycle	8.1	0.5	9.2	1.6	1.9	2.5	1.7

*The micronutrient concentrations were the following: Fe (20 μM), Mn (5 μM), Zn (2 μM), B (25 μM), Cu (0.5 μM), and Mo (0.1 μM). The starting EC value of the NS was 1.7 dS ⋅ m^-*1*^. The concentrations of Na^+^ and Cl^-^ increased until the values reported into the experimental protocol.*

The NS with the concentrations reported in **Table 1** was defined “full strength” NS, where the strength means the concentration of the nutrients per part of water, and it was assigned an arbitrary value of 1. During the crop cycle the strength of the NS used to replenish the consumption, when needed, was decreased from 1 to 0.5 or 0.33 in order to avoid the excessive increase of the EC due to the recycling of the NS inside the system.

When the NS in a treatment was replaced, a sample of the discharged solution was collected and analyzed to determine the concentration of the following elements: N (both from NO_3_^-^ and NH_4_^+^), H_2_PO_4_^-^-P, K^+^, SO_4_^2-^-S, Na^+^, Mg^2+^, and Ca^2+^.

##### Growth analysis, nitrogen, and cation uptakes

Fortnightly one plant from each experimental unit was harvested and used to measure the following parameters: fresh and dry weight of leaves, stem, and roots; number, fresh, and dry weight of fruits. After the plant removal, the remaining were rearranged in order to maintain the density of 3.3 plants⋅m^-2^.

The leaves removed with pruning and the harvested fruits were weighed for each individual experimental unit, dried in a forced draft oven and analyzed for N, P, K^+^, Ca^2+^, Mg^2+^, and Na^+^.

##### Crop Growth Rate (CGR) and crop transpiration–biomass ratio

The total biomass, transpiration, and mineral composition of the plant are required for the determination of the transpiration–biomass ratio. On the basis of the total dry weight of the plants (see “Growth Analysis, Nitrogen, and Cation Uptakes” section), calculated in correspondence with the fortnightly samples, the CGR was calculated as follows (Hunt, 1982):

$$C\,G\,R=\frac{\left(W_{2}-W_{1}\right)}{\left(t_{2}-t_{2}\right)}$$

Where, *W*_2_ and *W*_1_ are the dry weights of the plants at time *t*_2_ and *t*_1_, respectively.

Transpiration, which in a NFT system can be considered approximately equal to the water used by the plant, since there are no losses by evaporation, was calculated as outlined in “Nutrient Solution Management and Water Consumption” section, and was expressed on a daily basis for each interval of time between two destructive sampling of plants.

The transpiration–biomass ratio, calculated for the time unit, is the amount of water required (in grams) to produce the unit of dry matter (also in grams), and was calculated from the ratio between transpiration and CGR.

##### Determination of the nutrients levels for the recovery NS

For the determination of the recovery NS, we considered the concentration of the main elements (N, P, K^+^, Ca^2+^, Mg^2+^) and Na^+^ in the plant tissues and the transpiration of the crop. The concentration was determined for the several organs of the plant for every destructive sampling, leaf pruning and harvest. Since the different organs in plant account differently for DM percentage, element concentrations and absolute weight, the mineral composition of the plant was calculated by pondering the concentrations of the individual elements above reported of the various organs (stem, leaves from the destructive sampling and leaf pruning, roots, fruits from harvest and destructive sampling) to their respective dry weights.

The transpiration data and CGR were interpolated over time to calculate, through their respective functions, the transpiration–biomass ratio [see “Determination of Crop Growth Rate (CGR), Crop Transpiration, and Transpiration–Biomass Ratio” section].

Such data, together with the mineral composition of the plants for each phenological stage, weighted for the different organs of the plant, were inserted into spreadsheet software in order to calculate in real time, as a function of transpiration, the composition of recovery NS for the reintegration of nutrients and water removal from the plant. Finally, the concentration of each element in the plant was divided by the transpiration–biomass ratio to obtain the respective concentration (in mM) to be used in the recovery NS of the algorithm treatment.

#### Second Trial

The treatments, which had all the same starting EC, differed in the management of the NS and were the following (**Figure 1II**): (1) DS: the starting NS was Double Strength with respect to the full strength as defined in **Table 1**. The full strength was maintained up to the limit of 5 dS/m. From 5 to 7 dS/m the NS was full strength, from 7 to 9 dS/m it was ½ strength, and for values greater than 9 dS/m the NS used was 1/3 strength, according with the management in commercial greenhouses; (2) NaCl: the starting NS was full strength, and its EC was increased until the starting EC (3.2 dS/m) by adding NaCl to the NS. This strength was maintained up to the limit of 5 dS/m. Above this value the NS management was the same as the DS treatment and (3) TRASPBIO: the starting NS was the same as the DS treatment, until the EC value of 5 dS/m. When the EC value reached 5 dS/m, the NS was managed using the recovery solution which composition was defined in the first trial. The NS was not discharged into the environment in any treatment during the crop cycle.

### Statistical Analysis

The statistical analysis was performed with the Statistical Analysis System software (SAS, 1999) using the General Linear Model (GLM Proc; SAS9.1; SAS Institute, Cary, NC, USA) for the analysis of variance, the RGR procedure (regression) for the study of polynomial functions and the NLIN procedure for the study of non-linear functions.

For all morphological parameters, production, quality and chemical composition data, the comparison between the means point was performed by calculating the least significant difference (LSD, *P* = 0.05).

## Results

### First Trial

#### Nutrient Solution Management and Consumption

**FIGURE 2:**
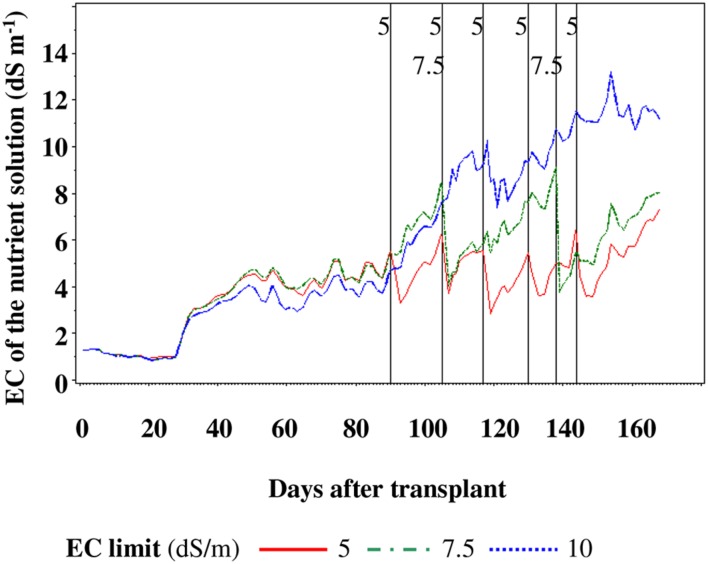
**Nutrient solution replacement in function of the reaching the electric conductivity (EC) limit.** Vertical bars indicate which EC limit was replaced at that time.

During the crop cycle, the NS was never replaced in SP10, while it was replaced five and two times for SP5 and SP7.5, respectively (**Figure 2**).

**FIGURE 3:**
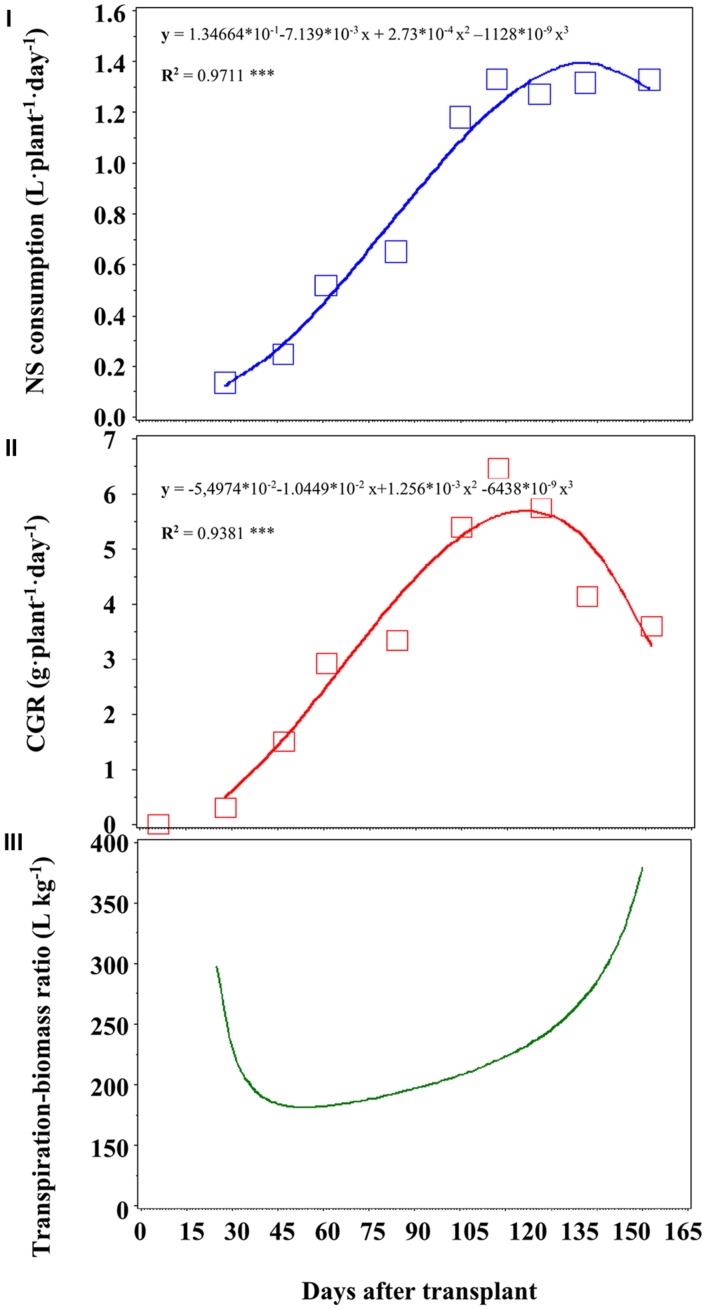
**Nutrient solution (NS) consumption (cumulated, I), daily production of biomass (CGR, II) and transpiration-biomass ratio (III).** ***P < 0.001.

The NS consumptions were similar in the three treatments and reached, on a daily basis and on average, 1.4 L⋅plant^-1^⋅day^-1^ 136 DAT (**Figure 3I**), while total water consumption at the end of the crop cycle was, on average, 137 L plant^-1^ (**Table 2**).

**Table 2:** Total yield, mean berry weight, percentage distribution of fruits into the diameter classes, and nutrient solution consumption (NSC) in function of the electric conductivity (EC) set point (SP) of the NS.

EC limit (SP; dS m^-1^)	Yield (kg plant^-1^)	Mean berry weight (g)	Number of berries 25–35 mm (%)	NSC (L plant^-1^)
SP5	3.184	21.0 a	56.8 b	139
SP7.5	2.942	19.5 b	75.5 a	133
SP10	3.035	19.1 b	76.4 a	139
Significance^1^	ns	*	***	ns

^1^Significance of F: ns = not significant for *P* ≤ 0.05; *P ≤ 0.05 and ***P ≤ 0.001, respectively. Different letters indicate statistically significant differences at *P* = 0.05.

#### Yield, Quality of the Fruits, and Plant Growth

The total yield was not influenced by treatments, with an average production of 3.054 kg plant^-1^ (**Table 2**). The fruit number per plant was unaffected by salinity (data not shown), while the number of fruits falling into the diameter class of 25–35 mm was, on average, 33% greater with the SP7.5 and SP10 treatments compared to SP5 treatment (**Table 2**).

**FIGURE 4:**
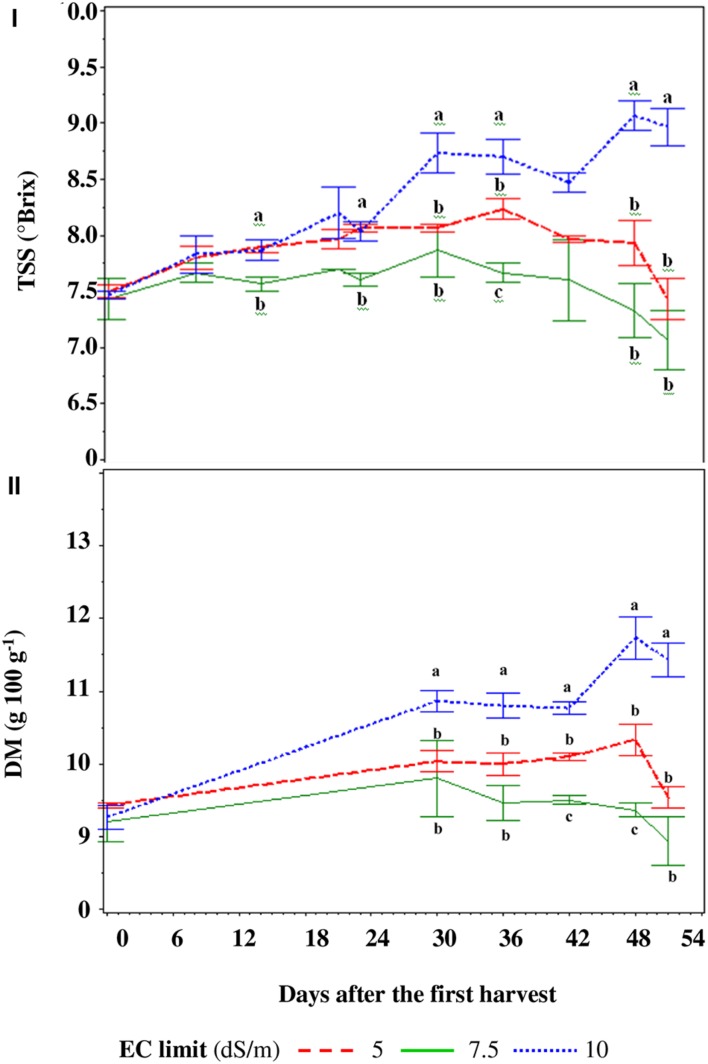
**Total soluble solids (TSS – I) and dry matter (DM – II) of the berries in function of the electric conductivity (EC) set point of the nutrient solution.** For every harvest, different letters indicate significant differences at *P* = 0.05.

The SP10 treatment showed a growing TSS trend during all harvest period while with the SP5 and SP7.5 treatments it increased until the fifth–sixth truss and then decreased (**Figure 4I**). The treatments did not show differences until the second truss, with an average value of TSS of 7.7°Brix, then the TSS content was highest in the SP10 treatment (with the exception of the third and fourth truss with respect to SP5 – **Figure 4I**), reaching a maximum value of 9.3°Brix (**Figure 4I**). Dry matter of the fruits showed a similar trend to the TSS values for all treatments and reached the maximum of 117 g kg^-1^ in SP10 (**Figure 4II**).

**FIGURE 5:**
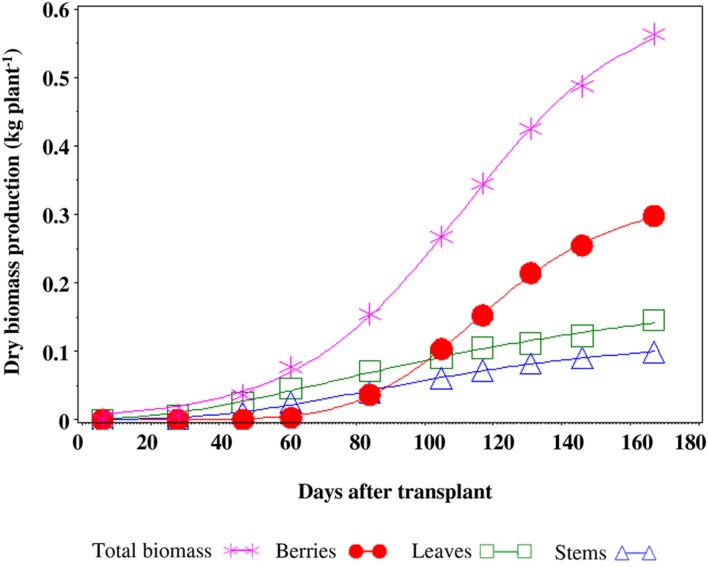
**Production of dry biomass and allocation in the different organs of a tomato plant**.

There were no differences between treatments for the dry biomass production of plants which amounted, on average, to 0.563 kg plant^-1^ (**Figure 5**). The total weight of fruits per plants (0.298 kg plant^-1^), accounted for 53% of the total dry biomass, while leaves and stems accounted for 28 and 19%, respectively (**Figure 5**).

#### Mineral Composition of the Canopy

**FIGURE 6:**
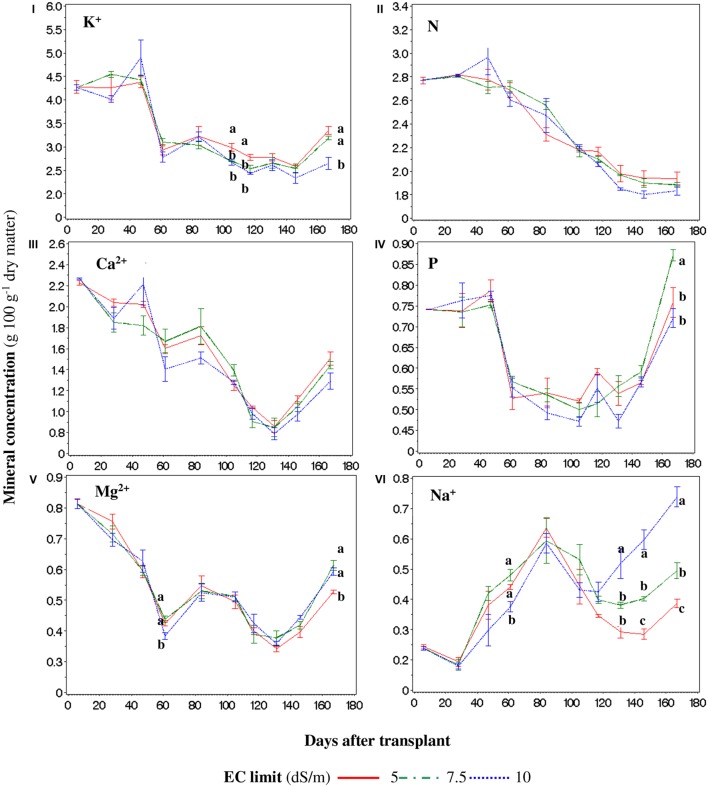
**Weighted concentrations for the whole plant of K^+^ (I), N (II), Ca^2+^ (III), P (IV), Mg^2+^ (V), and Na^+^ (VI) during the crop cycle in function of the set point limit of the electric conductivity (EC)**. For every point, different letters indicate significant differences at *P* = 0.05. If the letters are not present, there are no statistically significant differences between the means.

Considering the entire canopy (for space reason we consider the plant as the sum of leaves, stems, and fruits, and not the organs individually), the K^+^ concentration during the cycle was decreased by the SP7.5 and SP10 treatments 106 and 117 DAT, while at the end of the cycle only SP10 produced a lower K^+^ concentration (**Figure 6I**). The concentrations of K^+^ showed a decreasing trend in all treatments, but the lowest concentration was recorded in the SP10 100 DAT, mainly because of the lower concentrations in fruits and pruned leaves (data not shown). No differences between treatments were found for N and Ca^2+^ (**Figures 6II,III**), while for P, Mg^2+^, and Na^+^ occasional differences were recorded (**Figures 6I,IV–VI**, respectively). The highest concentration of Na^+^ was recorded in SP10 since the accumulation of NaCl in the NS (data not shown) caused an increase in Na^+^ concentration in all the plant tissues (**Figure 6VI**), especially in pruned leaves (data not shown). The tissue concentrations of Na^+^ reached lower levels (ranging from 3.8 to 7.4 g kg^-1^ – data not shown).

The P concentration showed a decreasing trend without differences between the treatments until 120–140 DAT; subsequently the concentration of P increased until 180 DAT (**Figure 6I**) where the SP7.5 showed a higher content.

The Mg^2+^ concentration (**Figure 6V**) was lower 61 DAT in the SP10 treatment (because of its concentration into the fruits – data not shown), and with SP5 at the end of the cycle (due to its concentration into the leaves – data not shown).

#### Recovery Solution: Meaning and Calculation

The recovery solution has its basis in the concept of “uptake concentrations” (Sonneveld and Voogt, 1997), and it is defined as the value of the amount of a nutrient removed by a crop divided by the volume of water absorbed in the same time interval, and is expressed in units of concentration (Sonneveld, 2000, 2002).

The consumption of NS reached a maximum of 1.4 L⋅plant^-1^⋅day^-1^ (**Figure 3I**).

We correlated the NS consumption (**Figure 3I**) with CGR (**Figure 3II**), and we obtained a curve describing the transpiration–biomass ratio (**Figure 3III**). Every point of such curve has a correspondent concentrations of a given nutrient into the recovery NS, which values are reported in **Table 3**.

**Table 3:** Nutrient levels into the recovery NS calculated in function of the real needs of the crop.

DAT	Phenological phases	N	K^+^	P	Ca^2+^	Mg^2+^	Na^+^	EC*
		(mM)						dS m^-1^
28	Beginning of flowering of first truss	8.0	4.4	1.0	1.9	1.2	0.3	1.2
47	Beginning of flowering of second truss	11.0	6.4	1.4	2.8	1.4	0.9	1.7
61	Beginning of flowering of fourth truss	10.5	4.1	1.0	2.1	0.9	1.0	1.3
84	Beginning of flowering of sixth – seventh truss + fruits	9.1	4.2	0.9	2.2	1.1	1.4	1.3
105	Beginning of flowering of 9th – 10th truss + fruits	7.5	3.4	0.8	1.6	1.0	1.0	1.1
117	First harvest	6.9	3.0	0.8	1.1	0.8	0.8	0.9
131	Second harvest	5.8	2.9	0.7	0.9	0.6	0.7	0.8
146	Sixth harvest	4.9	2.3	0.7	1.0	0.6	0.7	0.8
167	10th harvest	3.4	2.0	0.6	0.9	0.6	0.6	0.7

DAT, days after transplant. *Theoretical EC calculated using the formula proposed by Sonneveld and de Kreij (1999): EC = 0.095 + 0.19 *C*t, where *C*t is the total concentration of anions or cations present in the NS (in meq⋅L^-*1*^).

Observing the curve in **Figure 3III**, it is possible to divide it roughly into three different zones: in the first zone (approximately until 28 DAT) the transpiration–biomass ratio is quite high (about 300 L kg^-1^ dry weight), and later it tends to decrease with increasing of DAT (until 100 DAT). In this zone we can observe a rather stable phase, in which the concentration of the recovery NS should be higher than in the first zone of the curve. Finally, in the final part of the curve (after 100 DAT), the transpiration–biomass ratio increases again, up to almost 400 L kg^-1^ of dry weight.

#### Balance of Water and Nutrients: Input, Output WUE, and Nutrient Use Efficiency (NUE)

The input of water into the system was 14% greater in SP5 than in SP7.5 and SP10 (**Table 4**). Compared to SP5, for SP7.5 and SP10 the percentage savings of nutrients were, respectively: 20 and 25% (N), 21 and 27% (P), 19 and 27% (K^+^), 19 and 25% (Ca^2+^), 21 and 27% (Mg^2+^), 13 and 22% (S), 19 and 26% (total of all these minerals).

**Table 4:** Water and elements put into the NFT system in function of maximum electrical conductivity (EC) limit for the complete replacement of the NS.

EC limit (SP; dS m^-1^)	Water (m^3^ ha^-1^)	Nutrients (kg ha^-1^)					
		N	P	K^+^	Ca^2+^	Mg^2+^	SO_4_^2-^	Total
SP5	5,295 a	377 a	140 a	846 a	323 a	124 a	104 a	1.913 a
SP7.5	4,684 b	303 b	111 b	683 b	261 b	98 b	90 b	1.547 b
SP10	4,629 b	281 c	102 c	614 c	242 c	90 c	81 c	1.410 c
Significance^1^	**	***	***	***	***	***	***	***

^1^Significance of F: **P ≤ 0.01 and ***P ≤ 0.001. Different letters indicate statistically significant differences at *P* = 0.05.

The differences were even more marked for the quantities of water and elements removed from the system and discharged into the environment both during the cycle (only for SP5 and SP7.5) and at the end of it (for all the treatments – **Table 5**).

**Table 5:** Water and elements moved away from NFT system at the end of the cycle or during the cycle in function of maximum EC limit for the complete replacement of the NS.

EC limit (SP; dS m^-1^)	Water (m^3^ ha^-1^)	Nutrients (kg ha^-1^)								
		N	P	K^+^	Ca^2+^	Mg^2+^	SO_4_^2-^	Total	Cl^-^	Na^+^
SP5	842 a	182 a	57 a	292 a	204 a	196 a	207	1,138 a	1,146 a	568 a
SP7.5	420 b	74 b	25 b	120 b	169 a	176 a	181	745 b	821 ab	516 a
SP10	181 c	8 c	16 b	87 c	69 b	107 b	181	469 c	614 b	297 b
Significance^1^	***	***	***	***	***	**	ns	***	*	*

^1^Significance of F: ns, not significant for *P* ≤ 0.05; *P ≤ 0.05, **P ≤ 0.01, and ***P ≤ 0.001. Different letters indicate statistically significant differences at *P* = 0.05.

The SP10 allowed water savings of about 79 and 57% compared to SP5 and SP7.5, respectively (**Table 5**).

Considering the mineral savings, the SP7.5 and SP10 treatments allowed a saving of nutrients, with some considerations (**Table 5**): for N and K^+^, the savings, with respect to SP5, were, respectively, 59 and 96% (N) and 60 and 59% (K^+^). For P there was no difference between SP7.5 and SP10, so the discharged P, compared with SP5 was 64% on average. The differences for Ca^2+^, Mg^2+^, and Na^+^ varied between SP10 and the other treatments. With respect to SP5 and SP7.5, the saving of SP10 was, on average, 63, 43 and 45%, for Ca^2+^, Mg^2+^, and Na^+^, respectively; while for Cl^-^ the quantity of mineral discharged was lower only for SP10 compared to SP5 (46%).

For the total of the elements discharged, except for Na^+^ and Cl^-^, the savings with respect to SP5 were 35 and 59%, for SP7.5 and SP10, respectively (**Table 5**).

### Second Trial

#### Yield, Transpiration, and Water Use Efficiency (WUE)

The TRASPBIO treatment showed a greater yield with respect to other treatments, due primarily to bigger berries (**Table 6**).

**Table 6:** Total yield, mean berry weight and numbers of berries subdivided in diameter classes in function of the nutrient solution (NS) management, nutrient solution consumption (NSC).

NS management	Yield (kg plant^-1^)	Berry weight (g)	Number of berries (%)		NSC (L plant^-1^)
			25–35 mm	>35 mm	
TRASPBIO	3.606 a	20.6 a	50.5 b	47.0 a	167 a
DS	3.370 b	18.6 b	65.7 a	31.4 b	168 a
NaCl	2.916 c	18.1 b	77.0 a	20.4 b	146 b
Significance^1^	**	*	**	**	*

^1^Significance of F: *P ≤ 0.05 and **P ≤ 0.01, respectively. Different letters indicate statistically significant differences at P = 0.05.

The number of fruits falling into the class diameter of 25–35 mm was more frequent with the DF and NaCl treatments and was, on average, 41% greater than those of the TRASPBIO treatment (**Table 6**). This finding was reflected in the average weight of the fruits, which was 12% greater in the TRASPBIO treatment (**Table 6**).

The NaCl treatment showed the lowest transpiration (**Table 6**), but the WUE was not influenced by treatments, with a mean of 21 g/L (data not shown).

#### Quality of the Fruits

**FIGURE 7:**
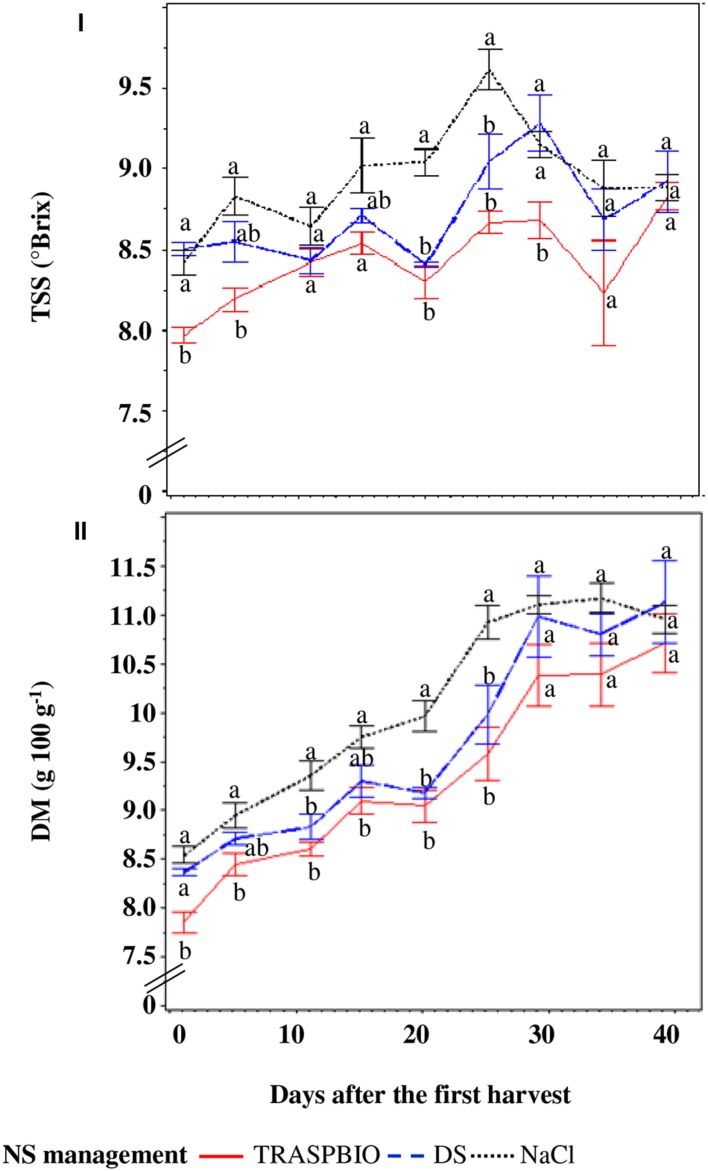
**Total soluble solids (TSS, I) and dry matter (DM, II) of the fruits in function of the nutrient solution (NS) management.** For every harvest, different letters indicate significant differences at *P* = 0.05.

The TSS content of berries was 5.4% greater in DF and NaCl than those of the TRASPBIO treatment (**Figure 7I**) and showed an increasing trend up to 30 days after the first harvest, then decreased.

The DM of berries of NaCl treatment was 7.9% higher than in the TRASPBIO treatment, with the exception of the last three harvests while, with respect to the DS treatment, it was only higher at three points (**Figure 7II**).

#### NS Consumption and Management

The NS was managed as shown in **Figure 1II** and was never replaced. Unlike in the first trial, NaCl treatment showed 13% lower plant transpiration with respect to DS and TRASPBIO.

#### Balance of Water and Nutrients: Input, Output, and Nutrient Use Efficiency (NUE)

Significant differences were observed for the NUE of all nutrients, with the exception of N (**Table 7**), with TRASPBIO treatment that produced the best NUE.

**Table 7:** Nutrients use efficiency (NUE) as a function of the NS management during the entire crop cycle.

NS management	NUE								
	Total	N	P	K^+^	Ca^2+^	Mg^2+^	SO_4_^2-^-S
	(kg yield/kg nutrient)								
TRASPBIO	46 a	180	633 a	110 a	260 a	806 a	776 a
DS	39 b	183	536 b	93 b	180 b	613 b	333 b
NaCl	40 b	186	546 b	93 b	186 b	623 b	350 b
Significance^1^	***	ns	**	**	***	***	***

^1^Significance of F: ns, not significant for *P* ≤ 0.05; **P ≤ 0.01 and ***P ≤ 0.001, respectively. Different letters indicate statistically significant differences at *P* = 0.05.

Less nutrients were moved away from the system in TRASPBIO (**Table 8**), with the exception of N and Na^+^ (higher concentrations) and P (no difference). Ca^2+^ and S accumulated more in the DS treatment, while TRASPBIO produced a saving of 48% for the K^+^ compared with DS and NaCl (**Table 8**). As expected, Na^+^ and Cl^-^ accumulated more in the NaCl treatment (**Table 8**).

**Table 8:** Water and nutrients moved away from NFT system at the end of the cycle in function of the NS management during the entire crop cycle.

NS management	Water (m^3^ ha^-1^)	N	P	K^+^	Ca^2+^	Mg^2+^	SO_4_^2-^-S	Total	Cl^-^	Na^+^
		(kg ha^-1^)								
TRASPBIO	108	49 a	7	31 b	18 b	19 b	6 c	130 b	24 c	32 b
DS	109	27 b	7	61 a	41 a	38 a	55 a	225 a	50 b	15 c
NaCl	108	16 b	6	57 a	22 b	27 ab	43 b	172 ab	72 a	43 a
Significance^1^	ns	**	ns	**	*	*	***	*	***	***

^1^Significance of F: ns, not significant for *P* ≤ 0.05; *P ≤ 0.05, **P ≤ 0.01, and ***P ≤ 0.001. Different letters indicate statistically significant differences at *P* = 0.05.

## Discussion

### First Trial

#### Yield, Quality of the Fruits, and Plant Growth

Total yield showed no significant differences between the salinity treatments (**Table 2**), with an average production of 3.054 kg plant^-1^, i.e., 10 kg m^-2^, a good production for a cherry tomato, considering the short period of production (less than 3 months, from May to July). This fact is not surprising, since salinity is a complex phenomenon acting on several aspects of plants. High salinity levels can result in inhibition of growth, smaller development of the plant and lower yield by way of osmotic stress, the injurious effects of toxic Na^+^ and Cl^-^ ions and nutrient imbalance caused by excess of these ions (Juan et al., 2005). However, such effects may be exacerbated (or mitigated) by several parameters (Hajer et al., 2006; Signore et al., 2008; Gent and Short, 2010, 2012; Komosa et al., 2011). The good yield performance of the SP7.5 and SP10 treatments was almost certainly due to the cultivation system used (NFT) and its influence on the hydraulic conductivity of plants. As reported by Savvas et al. (2007b), the irrigation frequency is of fundamental importance in maintaining optimal hydraulic conductivity, because it may maintain higher moisture levels in the substrates, increasing the unsaturated hydraulic conductivity and thus improving the availability of water at the root surface. Moreover, a high irrigation frequency, may improve crop performance due to a greater availability of nutrients (Silber et al., 2005). This fact is particularly true in the NFT system, where the availability of water (within the system and over time) is continuous and non-limiting. The fruit number per plant was unaffected by salinity (data not shown) similarly to Saito et al. (2006), while the number of fruits falling into the diameter class of 25–35 mm was, on average, 33% greater with the SP7.5 and SP10 treatments compared to SP5 treatment (**Table 2**), guaranteeing greater appreciation from the consumer (Serio et al., 2004; Signore et al., 2008).

The increase in TSS concentration over time was evident for the entire cycle for SP10 treatment (**Figure 4I**), while for SP7.5 and SP5 it reached a maximum then decreased. This behavior is most likely due to the accumulation of Na^+^ in the NS and its subsequent accumulation in the plant tissues (see below), since no difference was found for transpiration values. High EC levels are required in the root zone from the start of the crop cycle to improve fruit quality in hydroponic tomato crops, especially for cherry type, although the EC values reported are not constant among the various authors. Sonneveld and de Kreij (1999) suggest that EC levels of up to 3.7 dS m^-1^ are adequate in the root zone, while other Authors (Dorais et al., 2000) found that the target EC for tomato can be increased up to 40% above the recommended EC by Papadopoulos (1991) without compromising fruit yield. However, such a threshold value depends on cultivation parameters even with the same type of berry (Nakano et al., 2010). The DM content was higher in SP10 treatment, corroborating previous findings (Mori et al., 2008).

The total biomass production and the partitioning of DM into various organs were not affected by saline treatments, in agreement with Li and Stanghellini (2001). The fruit percentage represented 53% with respect to total biomass, and such data is consistent with previous findings (Hsiao, 1993).

Such results demonstrate that when the supply of assimilate is not limiting, the amount of assimilates being imported by a fruit is not affected by the water relation in the plant, according to Ehret and Ho (1986). The absorption of nutrients and transport of assimilates into the plant can be hindered at root and/or leaf level, because salinity can affect the absorption of water and nutrients by the roots and may decrease the leaf area development. When the canopy is incomplete, and only a part of PAR is intercepted, any factor that reduces the rate of canopy development would slow down the rate of biomass accumulation and, moreover, the effect of water stress on leaf growth tends to be compounded with time, leading to a larger reduction in biomass when compared to the reduction in relative growth rate (Hsiao, 1993). In our case the reduction in biomass accumulation did not occur because the source of assimilate, namely the canopy, did not show significant differences (data not shown). At root level the absorption of water and nutrients was not hindered by salinity, providing them with adequate transport to the leaves, and this may be ascribed to the level of EC reached in the NS at a particular growth stage. In fact, as reported by van Noordwijk (1990), the roots of tomato stop growing about 56 days after planting because of heavy fruit loads competing for carbohydrates, and this halt in root growth may reduce nutrient uptake, but not water uptake. In our case, at that development stage, the EC of the NS was approximately 4–4.5 dS m^-1^, without differences between treatments (**Figure 2**), so almost certainly there were no conditions that could have led to a different absorption of water or nutrients in the different treatments.

#### Nutrient Solution Consumption

The cumulative water consumption reached 137 L plant^-1^ in the first trial (**Table 2**), without differences between the treatments and, since evaporation and uncontrolled bleeding from NFT systems are negligible (Pardossi et al., 2005), the water consumption corresponded to crop water uptake driven by plant transpiration and growth. The lack of differences between the treatments is likely to be ascribed to the irrigation frequency that is not limiting in the NFT system, consistently with (Savvas et al., 2007b), who found that a lower irrigation frequency is associated with a lower cumulative water uptake at each level of irrigation water salinity. The NS was diluted (if necessary) to bring down the EC values, in particular during the months with the highest temperatures, when it could become difficult to maintain the target EC of the corresponding treatment which may have led to nutrient imbalance. Such management is a common practice in the closed soilless system (Hao and Papadopoulos, 2002; Magan et al., 2008; Komosa et al., 2011) and it is due to the domination of water transpiration over the uptake of nutrients by plants and the selective uptake of ions.

#### Mineral Composition of the Canopy

Considering the entire canopy (for space reason we consider the plant as the sum of leaves, stems, and fruits, and not the organs individually), the K^+^ concentration during the cycle was decreased by the SP7.5 and SP10 treatments 106 and 117 DAT, while at the end of the cycle only SP10 produced a lower K^+^ concentration (**Figure 6I**). The concentrations of K^+^ showed a decreasing trend in all treatments, but the lowest concentration was recorded in the SP10 100 DAT, mainly because of the lower concentrations in fruits and pruned leaves (data not shown). This result corroborates previous findings (Parra et al., 2007; Giuffrida et al., 2009) which indicate that a high concentration of Na^+^ may lead to a diminution of K^+^ and other macronutrients in plant tissue, especially in leaves (Cuartero and Fernández-Muñoz, 1999). However, previous studies regarding the K^+^ decrease in function of the salinity level provides contradictory results (Zekki et al., 1996; Komosa et al., 2011; Gent and Short, 2012). In our case, such a decrease was punctual and was probably due to two main reasons: primarily to the more frequent changes of NS in the SP5 treatment, unlike SP7.5 and SP10, which led to a greater concentration of the ion in the NS, allowing its better absorption. Secondly, the adaptation of the young plants to the salinity, which is reported as a process that allows plants to better uptake K^+^ and translocate it into the organs, in particular into the leaves (Parra et al., 2007). Since these differences between treatments were punctual, they did not affect the productive performance of the plants, also because they mainly occur at the end of the cycle, when most of the fruits were already set. This is an important aspect since maintenance of adequate K^+^ levels (and the resulting K/Na ratio), is essential for plant survival in saline habitats (Yurtseven et al., 2005), and in our case such ratio was scarcely affected until almost the end of the cycle (data not shown).

No differences between treatments were found for N and Ca^2+^ (**Figures 6II,III**), while for P, Mg^2+^, and Na^+^ occasional differences were recorded (**Figures 6I,IV–VI**, respectively). The highest concentration of Na^+^ was recorded in SP10 since the accumulation of NaCl in the NS (data not shown) caused an increase in Na^+^ concentration in all the plant tissues (**Figure 6VI**), especially in pruned leaves (data not shown) confirming previous findings (Giuffrida et al., 2009). The tissue concentrations of Na^+^ reached lower levels (ranging from 3.8 to 7.4 g kg^-1^ – data not shown) than those reported by Lovelli et al. (2012) despite the similar EC of the NS. This is probably due to the different typology of tomato and to the fact that in our experiment Na^+^ was mainly compartmentalized into the pruned leaves or into the root (data not shown). The partitioning into the roots can be explained as an effort by the plant to strengthen the ion detoxification capability of roots (Maggio et al., 2007), while its accumulation in leaves is due to its movement through the transpiration water flux, especially at high salinity levels, since at low salinity levels the Na^+^ is extruded from the cytoplasm into the apoplastic space (Shi et al., 2003) and/or compartmentalized into the vacuole (Blumwald et al., 2000; Zhang and Blumwald, 2001).

The notable differences in Na^+^ concentration, from 130 DAT until the end of the cycle, were most likely due to two main reasons: (i) the accumulation of Na^+^ in the NS of SP10 treatment (data not shown) because of continuous recycling and (ii) the periodic discharge of NS for SP5 and SP7.5 treatment, because of reaching the EC SP (**Figure 2**).

The P concentration showed a decreasing trend without differences between the treatments until 120–140 DAT; subsequently the concentration of P increased until 180 DAT (**Figure 6I**) where the SP7.5 showed a higher content.

The Mg^2+^ concentration (**Figure 6V**) was lower 61 DAT in the SP10 treatment (because of its concentration into the fruits – data not shown), and with SP5 at the end of the cycle (due to its concentration into the leaves – data not shown).

#### Recovery Solution Definition

The consumption of NS reached a maximum of 1.4 L⋅plant^-1^⋅day^-1^ (**Figure 3I**) a few days before of the maximum CGR (**Figure 3II**), since the mineral elements must be photosynthesized in order to be available. The curve in **Figure 3III** is composed by three different zones: in the first and last one, the transpiration–biomass is quite high, that means lower concentrations of the nutrients into the NS. In the middle zone, the transpiration–biomass ratio is lower, indicating that the crop needs a more concentrated recovery NS.

The increase in the transpiration–biomass ratio in the final stage is probably the sum of several factors. A first factor is the different behavior of photosynthesis and transpiration with increasing solar radiation. Indeed, from a certain point onward, photosynthesis stops or tends to decrease, while the transpiration continues to increase (Sonneveld, 2002). The trend reported in **Figure 3III** (and the corresponding concentrations in **Table 3**) suggests that in the first and last phase of the curve, the recovery NS may contain lower concentrations of nutrients, since the transpiration component assumes greater importance. In the middle part of the curve, the transpiration component is less prominent, so in this phase the recovery NS should be more concentrated. The concentrations of macronutrients (in mM) of recovery NS during the crop cycle, in function of several stages, are reported in **Table 3**. The nutrient concentrations until 28 DAT (namely, the first destructive sampling) match the first part of the curve, from 28 to 105 DAT the central phase, and those concentrations from 105 until the end of the crop the third part of the curve (**Figure 3III**).

Beyond the nutrient concentrations, in soilless systems it is important that the elements present in the NS are always available. This should lead to reducing the concentrations of macronutrients (Siddiqi and Kronzucker, 1998), especially of N, particularly close to the end of crop cycle (**Table 3**), in order to increase the NUE and to reduce the environmental impact of soilless systems (Le Bot et al., 2001).

#### Input and Output of Nutrients, WUE, and Nutrient Use Efficiency (NUE)

In well-watered crops, where water is not a limiting factor, the WUE can be increased in three main ways (Baille, 2001): (i) increasing the physiological efficiency and the transpiration; (ii) reducing the evaporative component (virtually absent with the NFT growing system) and (iii) reducing the loss of water due to the drainage recycling part or all of the NS. In order to keep the WUE as high as possible and at the same time trying to achieve the lowest nutrient emission, several nutrient management, and discharge strategies have been worked out (Voogt and Van Os, 2012). The most of the water (and nutrient) losses normally occur at the beginning of a cycle, since the proportion of applied water used by the crop increases as the crop develops (Grewal et al., 2011) and more than 80% of the applied water (containing nutrients) is drained off during the first four weeks of the growing period and that the reuse of drainage water satisfied one-third of the water requirements of the crop (Grewal et al., 2011).

Unlike other Authors (Grewal et al., 2011; Komosa et al., 2011; Meric et al., 2011; Gent and Short, 2012), who compared closed and open soilless systems, we compared a closed and semi-closed cycle, since we wanted to verify the best way to reduce the discharge of water and nutrients into the environment. Comparing the amounts of water moved away from the system to the amounts added in the first cycle, the percentages were 16, 9, and 4% for SP5, SP7.5, and SP10, respectively. This means that the SP10 treatment produced 75% lower drainage compared to SP5, and 56% lower than SP7.5. Regarding the NUE, SP10 always produced the best results (**Tables 4** and **5**) for all the parameters, with the exception of SO_4_^2-^-S that was moved away from system.

### Second Trial

#### Yield, Transpiration, and Water Use Efficiency (WUE)

The highest yield performance achieved in the TRASPBIO treatment (**Table 6**) is probably due to differences in the management of the NS. Indeed, from 40 DAT, the NS was managed according to the algorithm in the ALGO treatment, allowing more punctual management of nutrient concentrations. Since no differences were found for transpiration, the differences in yield performance can be explained by the higher concentrations of macronutrients in the DS treatment, because a high EC due to macronutrients may adversely affect vegetative growth (Dorais et al., 2000). The distribution of the berries in the 25–35 mm class was higher in DS and NaCl, confirming that a high EC level in the cherry type tomato is of fundamental importance in obtaining high quality fruits (Serio et al., 2004; Signore et al., 2008).

#### Nutrient Solution Consumption

NaCl treatment showed 13% lower plant transpiration with respect to DS and TRASPBIO treatments, and this different behavior was probably due to the higher initial value of EC of the NS in NaCl treatment. Indeed, in the first trial the starting EC was 1.7 dS/m, while in the second one it was 3.2 dS/m, and it is well known that transpiration decreases with increasing rates of NaCl (Cuartero and Fernández-Muñoz, 1999; Lee and Van Iersel, 2008).

#### Balance of Water and Nutrients: Input, Output, and Nutrient Use Efficiency (NUE)

The TRASPBIO treatment allowed a better NUE with respect to all the nutrients, with the exception of N (**Table 7**). Improving the NUE (together with WUE) is of a fundamental importance, since in the latest years soilless closed systems have gained popularity as a means to reduce water and fertilizer consumption (Neocleous and Savvas, 2016). With respect to the water and nutrients moved away from the system, TRASPBIO always showed the most notable saving of nutrients (**Table 8**), with the exception of N, probably because of the double concentration at the beginning of the crop cycle. With reference to the quantity of Ca^2+^ and Mg^2+^ discharged at the end of the cycle, TRASPBIO showed no differences with respect to NaCl treatment, but in comparison with DF, which had the same double concentration of macronutrients at the beginning of the cycle, TRASPBIO showed a greater saving. This is surely due to a better management of the NS, in particular in the final part of the cycle, when a more dilute NS is needed, according with our results in the first trial.

## Conclusion

Tomato is reported as a crop moderately tolerant to salinity. In fact, at least for cherry tomato in NFT systems, the production can be realized with high values of EC in the NS, up to (at least) 10 dS m^-1^, without detrimental effects for yield and/or quality of the berries. From a practical point of view, the results obtained with the TRASPBIO treatment (a more diluted NS in the first and last phase of the transpiration–biomass curve) would allow a great saving of water and nutrients resulting in a lower environmental impact and cost savings for the farmer.

## Author Contributions

AS: Substantial contributions to the conception or design of the work; Drafting the work; Final approval of the version to be published; Agreement to be accountable for all aspects of the work in ensuring that questions related to the accuracy or integrity of any part of the work are appropriately investigated and resolved. FS: interpretation of data; revised the article critically; Final approval of the version to be published; Agreement to be accountable for all aspects of the work in ensuring that questions related to the accuracy or integrity of any part of the work are appropriately investigated and resolved. PS: Substantial contributions to the conception or design of the work; Analysis and interpretation of data; Final approval of the version to be published; Agreement to be accountable for all aspects of the work in ensuring that questions related to the accuracy or integrity of any part of the work are appropriately investigated and resolved.

## Conflict of Interest Statement

The authors declare that the research was conducted in the absence of any commercial or financial relationships that could be construed as a potential conflict of interest.

## Footnotes

1 http://faostat3.fao.org
