# Supplementary material for: A Targeted Management of the Nutrient Solution in a Soilless Tomato Crop According to Plant Needs
Source: Front Plant Sci. 2016 Mar 30;7:391. doi: 10.3389/fpls.2016.00391 (PMC4876364; doi:10.3389/fpls.2016.00391)
Supplement: Supplementary file 1 [file Data_Sheet_1.DOCX]

**Transpiration-biomass ratio: theoretical basis and description**

The reasons for which we have defined a recovery nutrient solution are multiple. First of all several models have been developed for the management of the nutrient solution (NS) in closed soil-less systems, but generally they need a plethora of variables in order to work and only a few models are suitable for vegetables in greenhouse environments (Bacci *et al*., 2012). A second reason is due to the fact that the nutrients concentrations needed in Mediterranean conditions to compensate for plant uptake in closed hydroponic systems, are generally lower than those commonly used, which are based on Dutch recommendations (Savvas *et al.*, 2007) and, finally, in closed systems a leaching of nutrient solution is normally needed, due to i) salt accumulation, resulting from nutrients supplied in excess to crop demand (good quality water) or to NaCl (poor quality water), and ii) shifts in relative concentration of individual ions (Van Noordwijk, 1990). This occurs because in an ideal situation, the nutrient supply should be equal to the demand of the crop, to avoid depletion or accumulation but, in a real scenario, this is not the case.

Ben-Gal *et al*. (2003) found that, under several conditions and levels of environmental stress, a linear yield – transpiration relationship throughout the life span of a crop, which means that the nutrient uptake and water absorption are independent of the crop system. This fact implies that, in correspondence of a specific phenological stage of a crop, there is an average concentration of nutrients that can be determined experimentally. Such an average concentration defines the recovery NS, which is determined by the ratio of transpiration to growth: the first determines the rate of water removal, the latter the rate of nutrients removal (Bugbee, 2004).

The archetype of our recovery solution starts from the assumption that for every species there is a linear relationship between transpiration and biomass production and that such a relationship depends on the growth stage and growth conditions of the crop (Ben-gal and Shani, 2002). For example, under different conditions of availability of water, salinity and nutrients in three separate growth stages (flowering, fruit enlargement and harvesting) of a tomato crop, it is possible to describe three linear relationships between evapo-transpiration and biomass production (Bugbee, 2004).

Although the uptake concentration concept has no physiological basis (Gallardo *et al*., 2009), it is considered a useful guide for the optimal formulation of fertigation solutions (Sonneveld, 2000; Sonneveld, 2002).

Anyway, the concept of the uptake concentration alone is not enough to define the nutritional requirements of a crop, because the higher the dry matter produced by crop the greater are the nutritional requirements and the more likely the occurrence of nutritional imbalances.

The concentration of ions in the recovery NS is determined by the ratio of transpiration to growth, while the transpiration determines the rate of water removal and the growth determines the rate of nutrient removal (Bugbee, 2004).

As a consequence, the composition of the recovery NS must be defined according to the ratio between uptake concentration (Sonneveld, 2000; Sonneveld, 2002) and the growth of the crop. For example, if we assume that a particular crop has a ratio 300:1 (namely, 300 litres of water per kilogram of dry matter (Bugbee, 2004), and we need to calculate the K^+^ concentration in the recovery NS, if the K^+^ concentration into the plant, at that particular moment of the cycle culture, is equal e.g. to 4 g 100 g^-1^ of dry matter (K^+^ = 4%, i.e. 40 g kg^-1^ of dry matter), there must be 40 g of K^+^ in 300 L of solution of reintegration, namely 0.133 g L^-1^ of K^+^. Dividing 0.133 by the atomic weight of K^+^, the recovery NS must have a concentration of: 0.133/39.09 = 0.0034 moles L^-1^ of K^+^, that is 3.4 mM K^+^; this calculation must obviously be extended to all macro nutrients.

In our research we have considered the main macronutrients for plant life, namely N, P, K^+^, Ca^2+^ and Mg^2+^, and collected the data from a real crop cycle, so the information provided by the recovery solution can be extended to the above elements. In this manner, knowing the relationship between transpiration and growth of a crop (transpiration ratio) and the mineral composition of the plant at a given moment, is possible to calculate the composition of the recovery NS than can be used to restore the consumption of water and nutrients at different stages in a cherry tomato crop cycle in a closed soilless system.

**References**

Bacci, L, Battista P., and Rapi B. (2012). Evaluation and adaptation of TOMGRO model to Italian tomato protected crops. *New Zealand J. Crop Hort. Sci.* 40, 115–26. doi: 10.1080/01140671.2011.623706

Ben-gal, A., Karlberg, L., Jansson, P.E., Shani, U. (2003). Temporal robustness of linear relationships between production and transpiration. *Plant Soil*, 1, 211–18. doi: 10.1023/A:1023004024653

Ben-gal, A., Shani, U. (2002). A highly conductive drainage extension to control the lower boundary condition of lysimeters. *Plant Soil* 239, 9–17. doi: 10.1023/A:1014942024573

Bugbee, B. (2004). Nutrient management in recirculating hydroponic culture. *Acta Hortic*. 648, 99–112.

Gallardo, M., Thompson, R.B., Rodríguez, J.S., Rodríguez, F., Fernández, M.D., Sánchez, J.A., Magán, J.J. (2009). Simulation of transpiration, drainage, N uptake, nitrate leaching, and N uptake concentration in tomato grown in open substrate. *Agric. Water Manag.* 96, 1773–84. doi:10.1016/j.agwat.2009.07.013.

Savvas, D., Stamati, E., Tsirogiannis, I., Mantzos, N., Barouchas, P., Katsoulas, N., Kittas, C. (2007). Interactions between Salinity and Irrigation Frequency in Greenhouse Pepper Grown in Closed-Cycle Hydroponic Systems. *Agric. Water Manag.* 91, 102–11. doi:10.1016/j.agwat.2007.05.001.

Sonneveld, C. 2000. Effects of salinity on substrate grown vegetables and ornamentals in greenhouse horticulture. Wageningen: Wageningen Agricultural University. <http://library.wur.nl/WebQuery/clc/975662>

Sonneveld, C. (2002). Composition of Nutrient Solutions. In Hydroponic Production of Vegetables and Ornamentals, edited by H. Savvas, D., Passam, 179–210. Athens, Greece: Embryo Publications.

Van Noordwijk, M. (1990). Synchronisation of Supply and Demand Is Necessary to Increase Efficiency of Nutrient Use in Soilless Horticulture. In Plant Nutrition - Physiology and Applications, edited by M.L. van Beusichem, 525–31. Kluwer Academic.

Bacci, L, Battista P., and Rapi B. (2012). Evaluation and adaptation of TOMGRO model to Italian tomato protected crops. *New Zealand J. Crop Hort. Sci.* 40, 115–26. doi: 10.1080/01140671.2011.623706

Ben-gal, A., Karlberg, L., Jansson, P.E., Shani, U. (2003). Temporal robustness of linear relationships between production and transpiration. *Plant Soil*, 1, 211–18. doi: 10.1023/A:1023004024653

Ben-gal, A., Shani, U. (2002). A highly conductive drainage extension to control the lower boundary condition of lysimeters. *Plant Soil* 239, 9–17. doi: 10.1023/A:1014942024573

Bugbee, B. (2004). Nutrient management in recirculating hydroponic culture. *Acta Hortic*. 648, 99–112.

Gallardo, M., Thompson, R.B., Rodríguez, J.S., Rodríguez, F., Fernández, M.D., Sánchez, J.A., Magán, J.J. (2009). Simulation of transpiration, drainage, N uptake, nitrate leaching, and N uptake concentration in tomato grown in open substrate. *Agric. Water Manag.* 96, 1773–84. doi:10.1016/j.agwat.2009.07.013.

Savvas, D., Stamati, E., Tsirogiannis, I., Mantzos, N., Barouchas, P., Katsoulas, N., Kittas, C. (2007). Interactions between Salinity and Irrigation Frequency in Greenhouse Pepper Grown in Closed-Cycle Hydroponic Systems. *Agric. Water Manag.* 91, 102–11. doi:10.1016/j.agwat.2007.05.001.

Sonneveld, C. 2000. Effects of salinity on substrate grown vegetables and ornamentals in greenhouse horticulture. Wageningen: Wageningen Agricultural University. <http://library.wur.nl/WebQuery/clc/975662>

Sonneveld, C. (2002). Composition of Nutrient Solutions. In Hydroponic Production of Vegetables and Ornamentals, edited by H. Savvas, D., Passam, 179–210. Athens, Greece: Embryo Publications.

Van Noordwijk, M. (1990). Synchronisation of Supply and Demand Is Necessary to Increase Efficiency of Nutrient Use in Soilless Horticulture. In Plant Nutrition - Physiology and Applications, edited by M.L. van Beusichem, 525–31. Kluwer Academic.
